# Timing of GTP binding and hydrolysis by translation termination factor RF3

**DOI:** 10.1093/nar/gkt1095

**Published:** 2013-11-08

**Authors:** Frank Peske, Stephan Kuhlenkoetter, Marina V. Rodnina, Wolfgang Wintermeyer

**Affiliations:** Department of Physical Biochemistry, Max Planck Institute for Biophysical Chemistry, Am Fassberg 11, 37077 Göttingen, Germany

## Abstract

Protein synthesis in bacteria is terminated by release factors 1 or 2 (RF1/2), which, on recognition of a stop codon in the decoding site on the ribosome, promote the hydrolytic release of the polypeptide from the transfer RNA (tRNA). Subsequently, the dissociation of RF1/2 is accelerated by RF3, a guanosine triphosphatase (GTPase) that hydrolyzes GTP during the process. Here we show that—in contrast to a previous report—RF3 binds GTP and guanosine diphosphate (GDP) with comparable affinities. Furthermore, we find that RF3–GTP binds to the ribosome and hydrolyzes GTP independent of whether the P site contains peptidyl-tRNA (pre-termination state) or deacylated tRNA (post-termination state). RF3–GDP in either pre- or post-termination complexes readily exchanges GDP for GTP, and the exchange is accelerated when RF2 is present on the ribosome. Peptide release results in the stabilization of the RF3–GTP–ribosome complex, presumably due to the formation of the hybrid/rotated state of the ribosome, thereby promoting the dissociation of RF1/2. GTP hydrolysis by RF3 is virtually independent of the functional state of the ribosome and the presence of RF2, suggesting that RF3 acts as an unregulated ribosome-activated switch governed by its internal GTPase clock.

## INTRODUCTION

Important steps of translation on the ribosome in bacteria are controlled by guanosine triphosphatases (GTPases), including initiation factor IF2, elongation factors EF-Tu and EF-G and release factor RF3. These GTPases share the same binding site on the ribosome and are activated on interaction with the ribosome (1). In the termination phase of protein synthesis, release factor 1 or 2 (RF1/2) recognizes stop codons on the messenger RNA (mRNA) in the A site (2). On binding of RF1/2 to a stop codon, the universally conserved GGQ motif of RF1/2 reaches into the peptidyl transferase center (3) and promotes peptidyl-tRNA hydrolysis (4–7). RF3 accelerates the dissociation of RF1/2 from the ribosome after peptide release (8). Another function of RF3 has been observed in quality control during translation elongation where RF3 stimulated the hydrolysis of erroneous peptidyl-tRNA by RF1/2 (9,10).

Crystal structures of ribosome complexes with RF3 and the non-hydrolyzable GTP analog GDPNP (11,12) indicate that in these complexes the ribosome is present in the hybrid/rotated state, whereas the pre- or post-termination ribosome complexes with RF1/2 assume the classic non-rotated state (13–16). The structures of ribosome–RF3 complexes indicate that RF1/2 binding would be destabilized in the hybrid/rotated state due to steric clashes, explaining why RF3 promotes the release of RF1/2.

The role of GTP binding and hydrolysis for the function of RF3 has not been fully clarified yet. A model was proposed (17–19), in which RF3–guanosine diphosphate (GDP), but not RF3–GTP, binds to the pre-termination complex (PreTC), i.e. the ribosome with peptidyl-tRNA in the P site and RF1/2 bound to a termination codon in the A site. According to that model, ribosome binding of RF3 accelerates GDP dissociation and re-binding, but GTP can only bind following the RF1/2-induced peptide release forming the post-termination complex (PostTC). Further, GTP binding to RF3 results in the release of RF1/2 from the ribosome, which, in turn, induces GTP hydrolysis. Owing to the low stability of RF3–GDP on the ribosome, RF3–GDP dissociates from the ribosome, completing the functional cycle.

Important features of that model were based on non-equilibrium measurements of nucleotide binding to RF3, using a nitrocellulose filter binding assay, which has proven unreliable for kinetically unstable nucleotide complexes of translation factors (20). Furthermore, except for a few stopped-flow data on the dissociation of GDP from RF3 on the ribosome (21), information from time-resolved experiments is lacking so far. The aim of the present article was to re-examine the effect of RF1/2 on GDP–GTP exchange on RF3, to measure guanine nucleotide affinities for binding to free and ribosome-bound RF3 at equilibrium and to determine the timing of peptide release and GTP hydrolysis during termination.

## MATERIALS AND METHODS

### Materials

All experiments were performed in buffer A [20 mM HEPES-KOH, pH 7.5 (37°C), 100 mM KCl, 7 mM MgCl_2_] at 37°C. Ribosomes from *Escherichia coli* strain MRE600 (22), f[^3^H]Met-tRNA^fMet^, *E. coli* initiation factors IF1, IF2 and IF3 (23), RF1/2 (24) and mutant RF2(GGA) (18) were prepared as described. For the preparation of RF3, the gene coding for RF3 with an N-terminal His-tag was cloned into a pET30a vector and expressed in BL21(DE3) cells. After purification by Ni-NTA affinity chromatography, the protein was purified to homogeneity by fast performance liquid chromatography on a Resource Q column (gradient 0–500 mM KCl in 20 mM HEPES-KOH, pH 7.5). The mRNA (28 nt) containing the sequence AUGUAA (i.e. a start codon followed by a stop codon) was purchased from IBA GmbH (Goettingen, Germany); a control experiment was performed with a similar mRNA with a UUC codon (coding for Phe) inserted between start and stop codons. 2′-/3′-O-(N′-methylanthraniloyl)guanosine-5′-O-diphosphate (mantGDP) and -triphosphate (mantGTP) as well as GDPNP, mantGDPNP and GDPCP were purchased from Jena Bioscience and did not contain detectable levels of GTP or GDP (20).

For 70S initiation complex (70S IC) formation, ribosomes (1.5 µM) were incubated with IF1, IF2 and IF3 (1.8 µM each), f[^3^H]Met-tRNA^fMet^ (2.7 µM), mRNA (3 µM) and GTP (1 mM) in buffer A for 30 min at 37°C. The extent of f[^3^H]Met-tRNA^fMet^ binding (>95% relative to ribosomes) was determined by filtration on nitrocellulose filters that were dissolved in scintillation fluid (Quickzint 361, Zinsser Analytic) and counted in a liquid scintillation counter (Tricarb 3110 TR, PerkinElmer). Initiation complexes were purified by ultracentrifugation (Optima Max-XP ultracentrifuge, SW55Ti rotor, 55 000 rpm, 120 min, Beckman Coulter) through a 40% sucrose-cushion in buffer A containing 20 mM MgCl_2_. Pellets were re-suspended in buffer A, frozen in liquid nitrogen and stored at −80°C.

### Rapid kinetics

Rapid kinetic experiments were performed on an SX-20MV stopped-flow apparatus (Applied Photophysics, Leatherhead, UK). Experiments were performed by rapidly mixing equal volumes (60 µl) of reactants at 37°C. RF3–guanine nucleotide complex formation or dissociation was monitored by the fluorescence of mantGDP, mantGTP or mantGDPNP, which was excited by FRET from tryptophan in RF3. The excitation wavelength was 290 nm, and the fluorescence emission was measured after passing a cut-off filter (KV408; Schott, Mainz, Germany).

For RF3–mantGDP complex formation, RF3 was preincubated with a 10-fold excess of mantGDP. To induce the dissociation of mantGDP from RF3, the complex was rapidly mixed with the respective competing nucleotide (GDP, GTP or GDPNP) at a final concentration of 1 mM. In experiments with GTP or mantGTP, phosphoenolpyruvate and pyruvate kinase were added for regeneration of GTP (20). Time courses shown are averages of 3–10 individual experiments. Vacant ribosomes or PreTC was used at concentrations of 0.05 µM for turnover and of 0.7 µM for single-round experiments. To obtain PostTC, PreTC was treated with either puromycin (3 mM, 10 min at 37°C), RF2 (4 µM, 5 min at 37°C) or RF2(GGA) (4 µM, 90 min at 37°C), as indicated.

### Equilibrium titrations

RF3–GDP (0.5–4 µM) was mixed with mantGTP, mantGDP or mantGDPNP as indicated (Figure 3A). PreTC or PostTC (0.05 µM) was mixed with RF2(GGA) (2 µM) and RF3–mantGTP/GDP was prepared using a 2-fold excess as indicated (Figure 3). For competition titrations with unlabeled guanine nucleotides (Figure 3B), the RF3–mantGDP complex was formed (40 µM RF3, 400 µM mantGDP) and purified on a NAP5 column. Fluorescence titrations were performed in a FluoroLog 3 spectrofluorimeter (Horiba Scientific; Edison, NJ, USA). The fluorescence of the mant group was excited by FRET from tryptophan in RF3. The excitation wavelength was 290 nm, and the emission was measured at 445 nm. The fluorescence change due to complex formation, Δ*F*, was determined by subtracting the signals obtained in a control titration of mant-labeled nucleotide in buffer without RF3 from the signals obtained in the presence of RF3 and its complex partners, if present. Difference plots were evaluated using the following quadratic equation, accounting for the concentration change of added ligand due to complex formation,

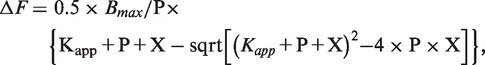

where *B*_max_ represents the amplitude, P the total concentration of RF3, X the total concentration of nucleotide and *K_app_* the apparent dissociation constant (see later). To measure the amount of GDP bound to RF3 after purification, the factor was incubated with ethylenediaminetetraacetic acid, and the released nucleotide was determined by ion exchange chromatography. Nucleotide-free RF3 prepared by the same method showed a decreased activity. Therefore, RF3, which had GDP bound in a 1:1 ratio, was used for the experiments. In the equilibrium titrations, the presence of GDP as a competitor was taken into account by recalculating the equilibrium constant using the following equation:

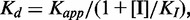

where *K_app_* is the apparent dissociation constant obtained from the titration curve without taking into account the presence of GDP, [I] is the concentration of GDP and *K_I_* is the dissociation constant of GDP estimated to 5 nM (17). To compare the affinities to RF3 of mantGDP or mantGTP with that of GDP, the results were re-plotted (Figure 3) according to the following equation, which does not require the knowledge of the exact K_I_ value:

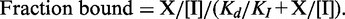


In this type of re-plot, half-saturation of the fit shows the K_d_/K_I_ ratio. The equation is derived on the assumption that K_I_/[I] is close to 0, which is plausible given the low reported *K_I_* value (5 nM) compared with the GDP concentrations used (2–4 µM).

### GTPase assay

Ribosomes or ribosome–RF2 complexes (2 µM) were mixed with RF3 (2 μM) in buffer A with GTP and trace amounts of [γ-^32^P]GTP at 37°C. Samples were quenched with one volume of 40% formic acid and analyzed by thin layer chromatography (Polygram CEL 300, Macherey–Nagel) using 0.5 M potassium phosphate (pH 3.5) as running buffer. Radioactivity was determined using a phosphoimager system.

## RESULTS

### Kinetics of GDP dissociation from free RF3 and ribosome-bound RF3

**Figure 1.**
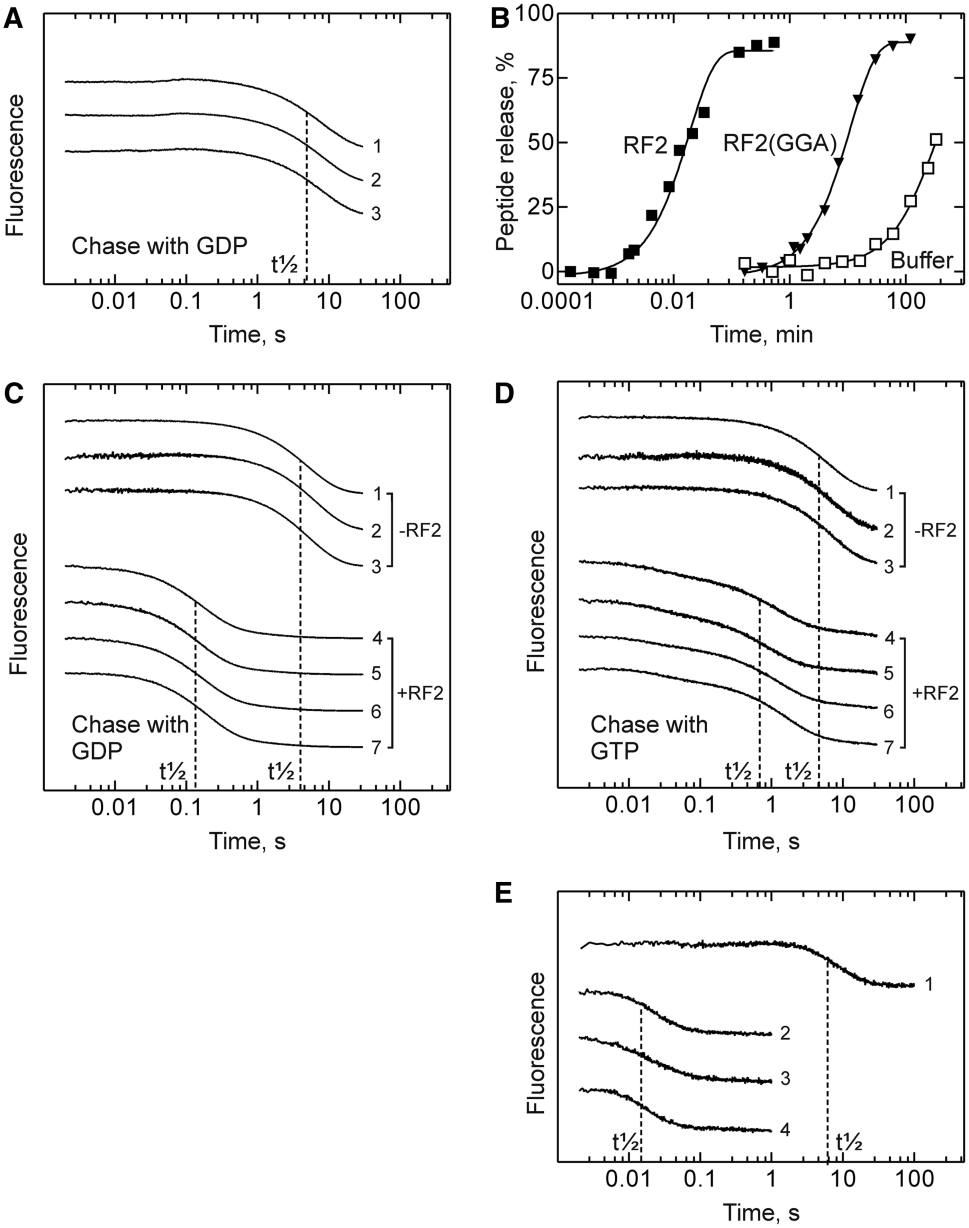
Kinetic stability of RF3–GDP complexes. For clarity, the overlapping curves are shifted vertically. Dashed lines indicate approximate half-life times (t_1/2_) of the respective complexes. (**A**) Dissociation of mantGDP from RF3–mantGDP (1), from RF3–mantGDP (1 µM) in the presence of RF2 (1 µM) (2), from RF3–mantGDP in the presence of RF2 (5 µM) (3), as induced by chase with GDP. (**B**) Peptide release activity of RF2 and RF2(GGA). The release activity was measured with pre-TC (0.25 µM) in buffer A at 37°C and RF2 (dark square; k = 0.9 ± 0.1 s^−1^) or RF2(GGA) [dark triangle; k = (1.5 ± 0.1) × 10^−3 ^s^−1^] (4 µM); residual f[^3^H]Met-tRNA^fMet^ was determined by TCA precipitation. As a control, the hydrolysis of free fMet-tRNA^fMet^ in the absence of RF2 is shown [buffer, light square; k = (6 ± 2) × 10^−5 ^s^−1^]. (**C**) Dissociation of mantGDP from RF3 (1 µM) on various ribosome complexes as induced by chase with GDP at conditions of RF3 turnover. The RF3–mantGDP complex was rapidly mixed with the PreTC and GDP (1); RF3–mantGDP versus PostTC (obtained by puromycin treatment) and GDP (2); RF3–mantGDP plus puromycin versus PreTC and GDP (3); RF3–mantGDP versus PreTC plus RF2(GGA) and GDP (4); RF3–mantGDP versus PostTC obtained by pre-incubation with RF2(GGA) and GDP (5); RF3–mantGDP versus PostTC plus RF2 and GDP (6); RF3–mantGDP plus RF2 versus PreTC and GDP (7). (**D**) Dissociation of mantGDP from RF3 bound to ribosomes as induced by chase with GTP at conditions of RF3 turnover. Traces are numbered as in (C). (**E**) Dissociation of mantGDP from RF3 (0.5 µM) in the presence of excess ribosomes (0.7 µM, single round conditions). The RF3–mantGDP complex plus RF2(GGA) was rapidly mixed with vacant ribosomes and GTP (1); RF3–mantGDP plus RF2(GGA) versus PreTC and GTP (2); RF3–mantGDP versus RF2(GGA), PostTC and GTP (3); RF3–mantGDP plus RF2(GGA) versus PreTC and GDP (4). Dissociation rate constants were 0.15 ± 0.05 s^−1^ (trace 1) and 35 ± 5 s^−1^ (traces 2–4).

To measure the kinetics of nucleotide dissociation directly, we made use of a fluorescent derivative of GDP, mantGDP, carrying the N′-methylanthraniloyl (mant) group at the 2′/3′ position, which does not affect the function of GDP or GTP on other translational GTPases (20,25). We rapidly mixed either free RF3–mantGDP or RF3–mantGDP bound to different ribosome complexes with excess unlabeled GDP or GTP, monitoring the fluorescence of the mant group excited by FRET from tryptophan in RF3 (Materials and Methods). The rate constant of mantGDP dissociation from unbound RF3 was 0.13 ± 0.01 s^−^^1^ and, as expected, was not influenced by the presence of RF2 (Figure 1A). To suppress peptidyl-tRNA hydrolysis by RF2 bound to the PreTC, we used mutant RF2(GGA) in which Gln in the GGQ motif was replaced with Ala (18). The mutation reduced the activity in peptide release to 0.0015 s^−^^1^ at saturation with the factor (Figure 1B). When the PreTC was to be studied, samples were not incubated with mutant RF2(GGA) for longer than ∼1 min, the time at which peptidyl-tRNA hydrolysis was still negligible. On the other hand, the residual activity of the mutant factor allowed for control experiments with PostTC−RF2 complexes obtained by prolonged incubation of PreTC with RF2(GGA). To avoid any potential long-term effect of RF3 incubations with the ribosome (e.g. exchange with GTP and GTP hydrolysis), RF3–mantGDP was kept in one syringe and ribosomes and excess unlabeled nucleotide (GDP or GTP) in the other. On mixing, RF3 was expected to bind to the ribosome very rapidly, similarly to other translational GTPases, such as EF-Tu and EF-G, for which the bimolecular binding constant was >10^7 ^M^−^^1 ^s^−^^1^ (26,27). The rate constant of mantGDP dissociation from ribosome-bound RF3, ∼0.15 s^−^^1^, was not much different from that of mantGDP dissociation from unbound RF3, but was strongly increased, even under conditions of RF3 turnover, when RF2 was present (Figure 1C). The acceleration was independent of the functional state of the ribosome, i.e. it was the same on vacant ribosomes, PreTC or PostTC. These results support the previous observation (18) that the presence of RF2 on the ribosome accelerates the dissociation of GDP from ribosome-bound RF3, independent of the functional state of the ribosome.

When we used GTP as a competitor under turnover conditions for RF3, we observed similar kinetics of mantGDP dissociation from RF3 on various ribosome complexes, both in the absence and presence of RF2 (Figure 1D). (The multiphasic character of the dissociation time courses is attributed to the rapid binding of GTP to RF3 under these conditions; this is addressed later.) The efficient chase by GTP of mantGDP from RF3 bound to PreTC implies that GTP does bind to RF3 on a PreTC containing peptidyl-tRNA in the P site, in contrast to a previous report (17).

To determine rate constants of GDP dissociation from RF3, chase experiments were also conducted at conditions of single turnover, i.e. in excess of ribosomes over RF3 (Figure 1E). The dissociation of mantGDP from RF3 bound to vacant ribosomes in the presence of RF2(GGA) (k_off_ ≈ 0.15 s^−^^1^) was strongly accelerated on both PreTC and PostTC (k_off_ ≈ 35 s^−^^1^), i.e. when RF2(GGA) was bound to a stop codon. GDP and GTP were equally efficient in inducing the dissociation of the complexes, in keeping with the notion that both GDP and GTP could bind to RF3 bound to the PreTC.

### Kinetics of guanine nucleotide binding to free and ribosome-bound RF3

**Figure 2.**
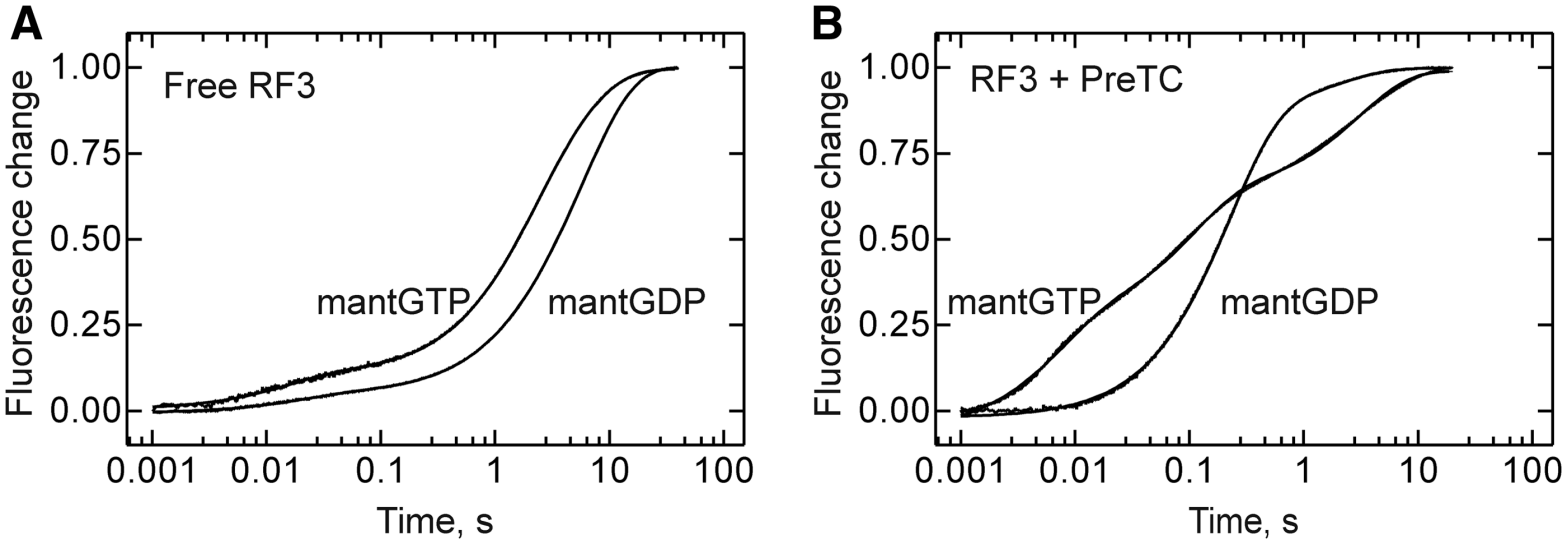
Time courses of GDP/GTP binding to free or ribosome-bound RF3. (**A**) Binding of mantGDP/mantGTP (10 µM) to free RF3 (1 µM). Time courses were evaluated by two-exponential fitting: mantGTP, k_app1_ = 34 ± 5 s^−1^, k_app2_ = 0.35 ± 0.01 s^−1^ and mantGDP, k_app1_ = 22 ± 2 s^−1^, k_app2_ = 0.18 ± 0.01 s^−1^. (**B**) Binding of mantGDP/mantGTP (10 µM) to RF3 (1 µM) bound to PreTC (0.05 µM) in the presence of RF2(GGA) (4 µM). The time course of mantGTP binding was evaluated by three-exponential fitting: k_app1_ = 130 ± 2 s^−1^, k_app2_ = 9 ± 1 s^−1^ and k_app3_ = 0.3 ± 0.1 s^−1^, and the time course of mantGDP binding by two-exponential fitting: k_app1_ = 4.5 ± 0.1 s^−1^ and k_app2_ = 0.5 ± 0.1 s^−1^.

To examine GDP and GTP binding to RF3 directly, we performed stopped-flow experiments with mant-labeled nucleotides, again monitoring the fluorescence of the mant group excited by FRET from tryptophan. Because attempts to prepare nucleotide-free RF3 resulted in protein inactivation, RF3 that contained an equimolar amount of GDP was used (Materials and Methods). When we rapidly mixed free RF3 with mantGDP or mantGTP, we observed a similar two-exponential binding behavior (Figure 2A). The major amplitude (>90%) was due to a slow phase that probably reflected the dissociation of GDP that was bound to a major fraction of RF3 and limited the rate of labeled nucleotide binding to ∼0.2 s^−^^1^ (mantGDP) or 0.35 s^−^^1^ (mantGTP), in agreement with the observed dissociation rate (Figure 1A). The rapid phase of ∼20–30 s^−^^1^ had a small amplitude and was probably due to a fraction of the protein, which could exchange the nucleotide rapidly. When RF3 was bound to a PreTC in the presence of mutant RF2(GGA), the binding of mantGDP was accelerated compared with the binding to unbound RF3 (Figure 2B). The time course of binding again was biphasic with a rapid phase of ∼4.5 s^−^^1^ (87% of the amplitude) and a slower phase of ∼0.5 s^−^^1^. The two phases may reflect a two-step binding mechanism or, alternatively, the dissociation of pre-bound GDP from ribosome-bound (∼6 s^−^^1^, Figure 1B) and unbound (0.15 s^−^^1^; Figures 1A and 2A) RF3, respectively, which limits the rate of mantGDP binding. Most importantly, the analogous experiment with mantGTP showed rapid binding of mantGTP to RF3 on the PreTC. The time course was triphasic, with apparent rate constants ∼130 s^−^^1^, 9 s^−^^1^ and 0.3 s^−^^1^. Although we have not analyzed the kinetics quantitatively, the experiment clearly shows, in accordance with the chase experiment presented above, that mantGTP binds to RF3 on a PreTC, and the binding kinetics indicates that GTP may bind faster than GDP. Thus, our measurements do not confirm the previous suggestion that the binding of GTP to ribosome-bound RF3 was precluded in the pre-termination state of the ribosome, whereas GDP dissociated and bound rapidly, independent of the functional state of the ribosome (17,18). In contrast, our kinetic data suggest that both GDP and GTP can bind to RF3 on either Pre- or PostTC.

### Affinities of GDP/GTP binding to RF3

**Figure 3.**
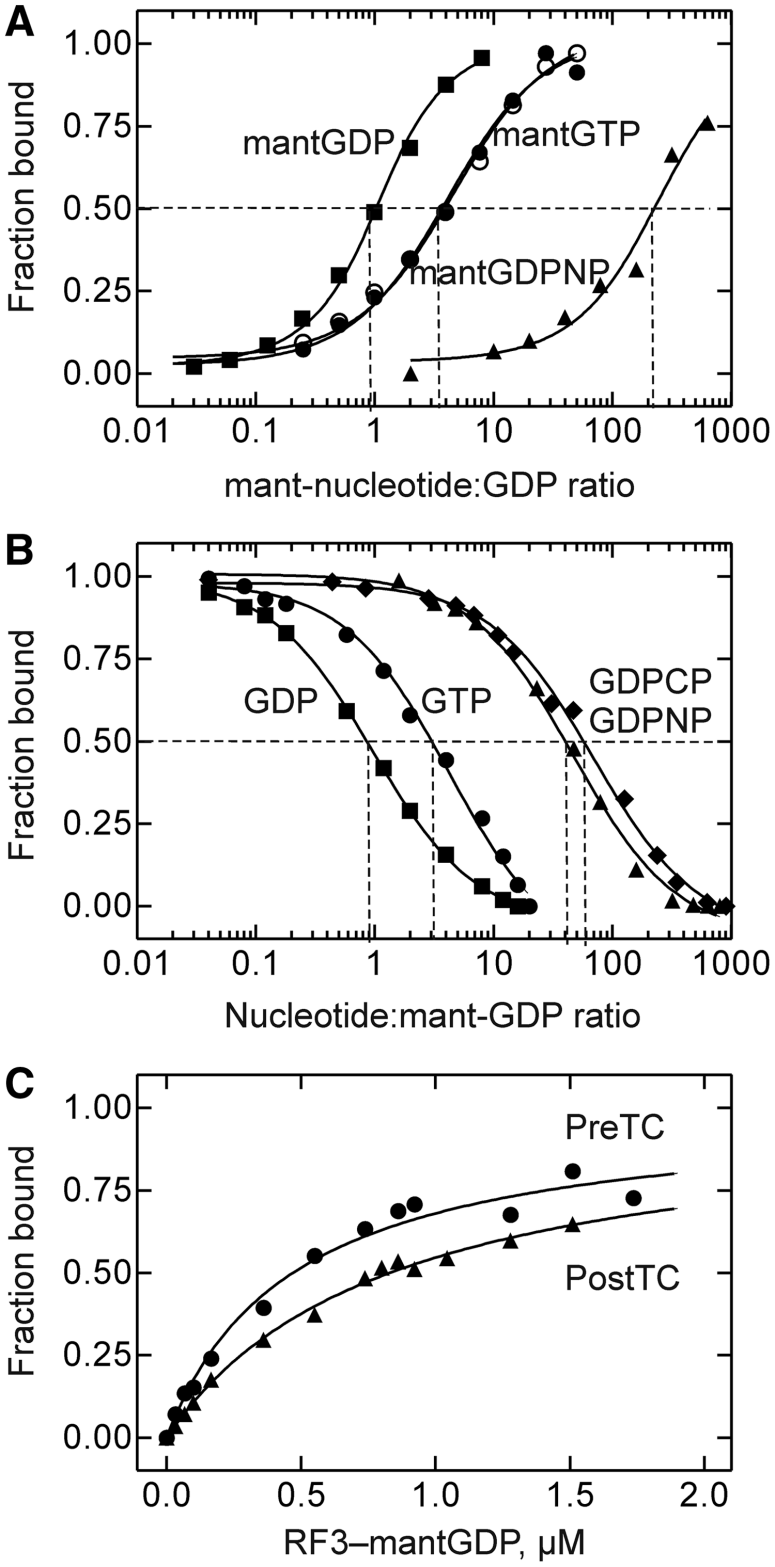
Equilibrium titrations. (**A**) Relative affinity of mant-labeled guanine nucleotide binding to RF3. The ratios of added mant-nucleotide:GDP at half-saturation are indicated (dashed lines). Titrations with mantGTP were performed without (dark circle) or with (light circle) RF2. (**B**) Competition titrations. The RF3-mantGDP complex, purified by gel filtration (Methods), was titrated with guanine nucleotides, as indicated, monitoring the fluorescence of mantGDP. The ratios of added nucleotide relative to mantGDP at half-saturation with GDP (1:1), GTP (3:1), GDPNP (40:1) and GDPCP (60:1) are indicated (dashed lines). (**C**) RF3–mantGDP binding to the ribosome. The preformed RF3–mantGDP complex was titrated against a PreTC (apparent K_d_ = 0.5 ± 0.1 µM) or PostTC obtained by incubation of PreTC with RF2 (apparent K_d_ = 0.8 ± 0.1 µM).

Previously, the affinities of guanine nucleotide binding to RF3 were determined by nitrocellulose filtration (17,18), a non-equilibrium technique that is problematic for the quantification of kinetically unstable factor-nucleotide complexes (20). Therefore, we have re-determined the affinities of GDP/GTP binding to RF3 by performing equilibrium titrations monitoring FRET between tryptophan of RF3 and the mant group (Figure 3). The evaluation of the titration curves had to take into account the presence of close to one GDP molecule in RF3. Thus, relative rather than absolute affinities were determined. The titration data were re-plotted to estimate the ratio between the K_d_ values for the respective mant-labeled nucleotide and unmodified GDP (Materials and Methods). For mantGDP, half-saturation was reached at a 1:1 ratio of mantGDP to GDP, indicating identical K_d_ values for mantGDP and GDP (Figure 3A). Assuming a K_d_ value for GDP of 5 nM, as obtained by nitrocellulose filtration (17), which is probably valid given the reasonable kinetic stability of the complex, the affinity of mantGDP is also 5 nM. In the titration with mantGTP, the ratio of added mantGTP and GDP at half-saturation was about 4 (Figure 3A); based on a K_d_ value of 5 nM for GDP, the K_d_ for mantGTP would be 20 nM, i.e. much lower than the reported value of 2.5 µM (17). Taking into account that the concentration of GTP in the cell is at least 10 times higher than that of GDP (28), the 4-fold affinity difference between the two nucleotides implies that RF3 in the cell is predominantly (>70%) present in the GTP-bound form. The K_d_ for mantGDPNP (Figure 3A) was >200-fold higher than the K_d_ for GDP. When the competition between unlabeled nucleotides and mantGDP was measured, we observed weaker binding also for unlabeled GDPNP (∼40-fold), and another non-hydrolyzable GTP analog, GDPCP (∼60-fold) (Figure 3B). Thus, the mant group weakened the binding of GDPNP to RF3 somewhat, in contrast to GDP or GTP, for which no such influence was observed. Weaker binding of GDPNP compared with GTP was observed previously for EF-G, although the difference was smaller [about 4-fold; (20)]. The affinity of RF3–mantGDP binding to Pre- and PostTC was found to be similar with K_d_ values of 0.5 µM and 0.8 µM, respectively (Figure 3C).

### Stabilization of the RF3–GDPNP complex on the ribosome

**Figure 4.**
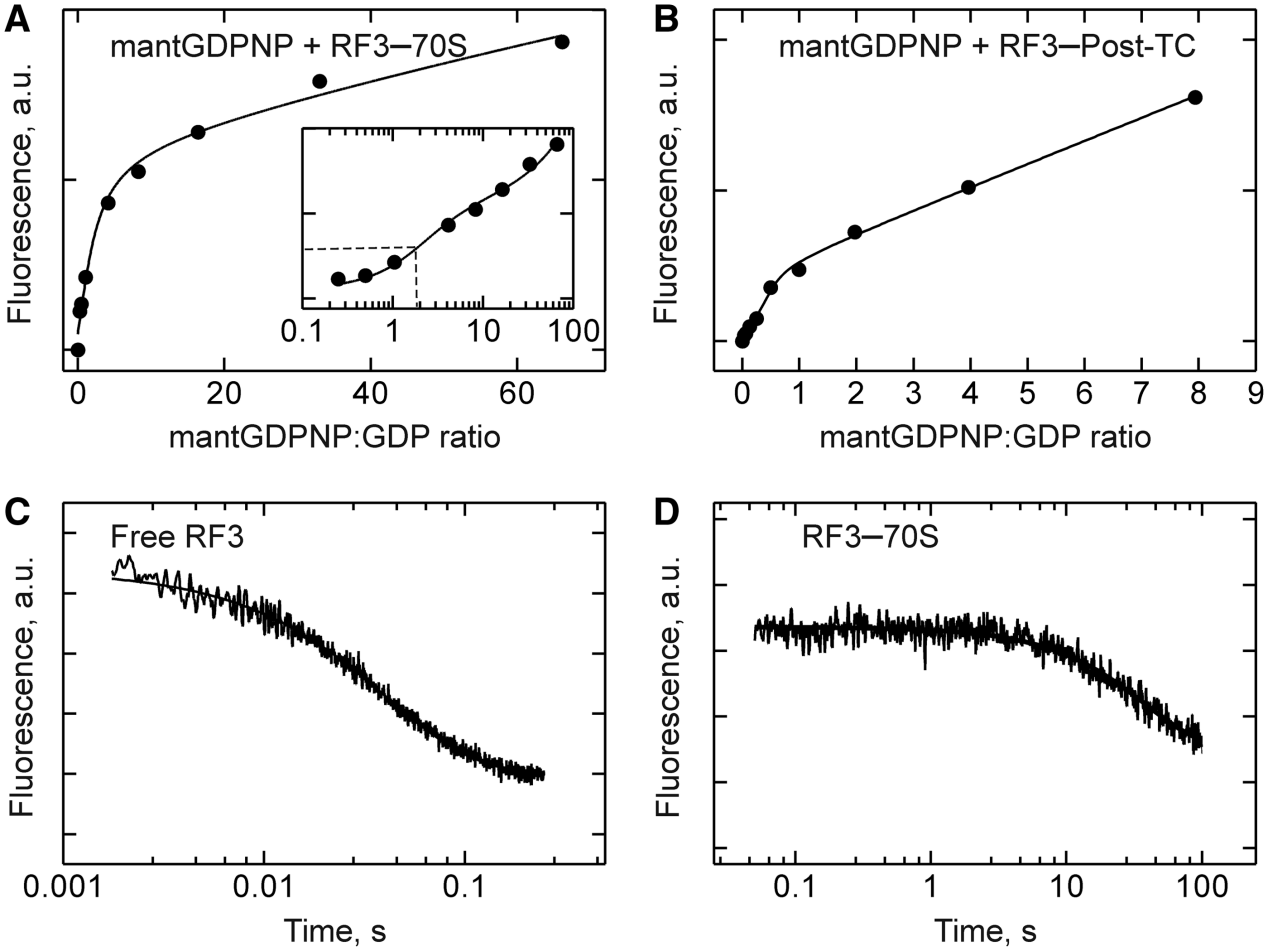
Stabilization of the RF3–GDPNP complex on the ribosome. (**A**) Equilibrium titration of mantGDPNP binding to RF3 (0.25 µM) bound to vacant ribosomes (2 µM). Inset: logarithmic plot, half-saturation of high-affinity binding is indicated (dashed lines). (**B**) Equilibrium titration of mantGDPNP binding to RF3 (2 µM) in the presence of PostTC (0.25 µM) and RF2 (4 µM). In control titrations without RF3, no signal change was observed. (**C**) Dissociation of mantGDPNP from free RF3 as induced by rapid mixing with excess unlabeled GDPNP; single-exponential fitting yielded k_off_ = 24 ± 1 s^−1^. (**D**) Dissociation of mantGDPNP (20 µM added) from RF3 (0.125 µM) bound to ribosomes (2 µM) as induced by adding excess unlabeled GDPNP (2 mM); k_off_ = 0.025 ± 0.001 s^−1^.

The affinity of GTP binding to ribosome-bound RF3 cannot be measured because in that complex GTP is hydrolyzed rapidly. To obtain an estimate for the affinity, we have used the non-hydrolyzable analog mantGDPNP and measured its binding to RF3 on vacant ribosomes (Figure 4A) or on PostTC (Figure 4B). Titrations were biphasic, yielding two equilibrium binding constants that were similar for the two complexes. The high K_d2_ value was similar to that measured for the RF3–mantGDPNP complex and, therefore, probably reflected nucleotide binding to RF3 that was not bound to ribosomes. The lower K_d_ values we attribute to the binding of mantGDPNP to ribosome-bound RF3, indicating an affinity increase of ∼1000-fold due to RF3 binding to the ribosome. The strong stabilization was entirely due to a lowered dissociation rate constant, which decreased by 1000-fold, from 24 s^−^^1^ for unbound RF3 (Figure 4C) to 0.025 s^−^^1^ for ribosome-bound RF3 (Figure 4D). The binding of GTP presumably is stabilized as well, but the effect cannot be determined directly because GTP would be hydrolyzed by ribosome-bound RF3. A strong stabilization of the GTP-bound (or GDPNP-bound) form of a translation factor on the ribosome has been observed previously for EF-G (20).

### GTP hydrolysis by RF3 on PreTC and PostTC

**Figure 5.**
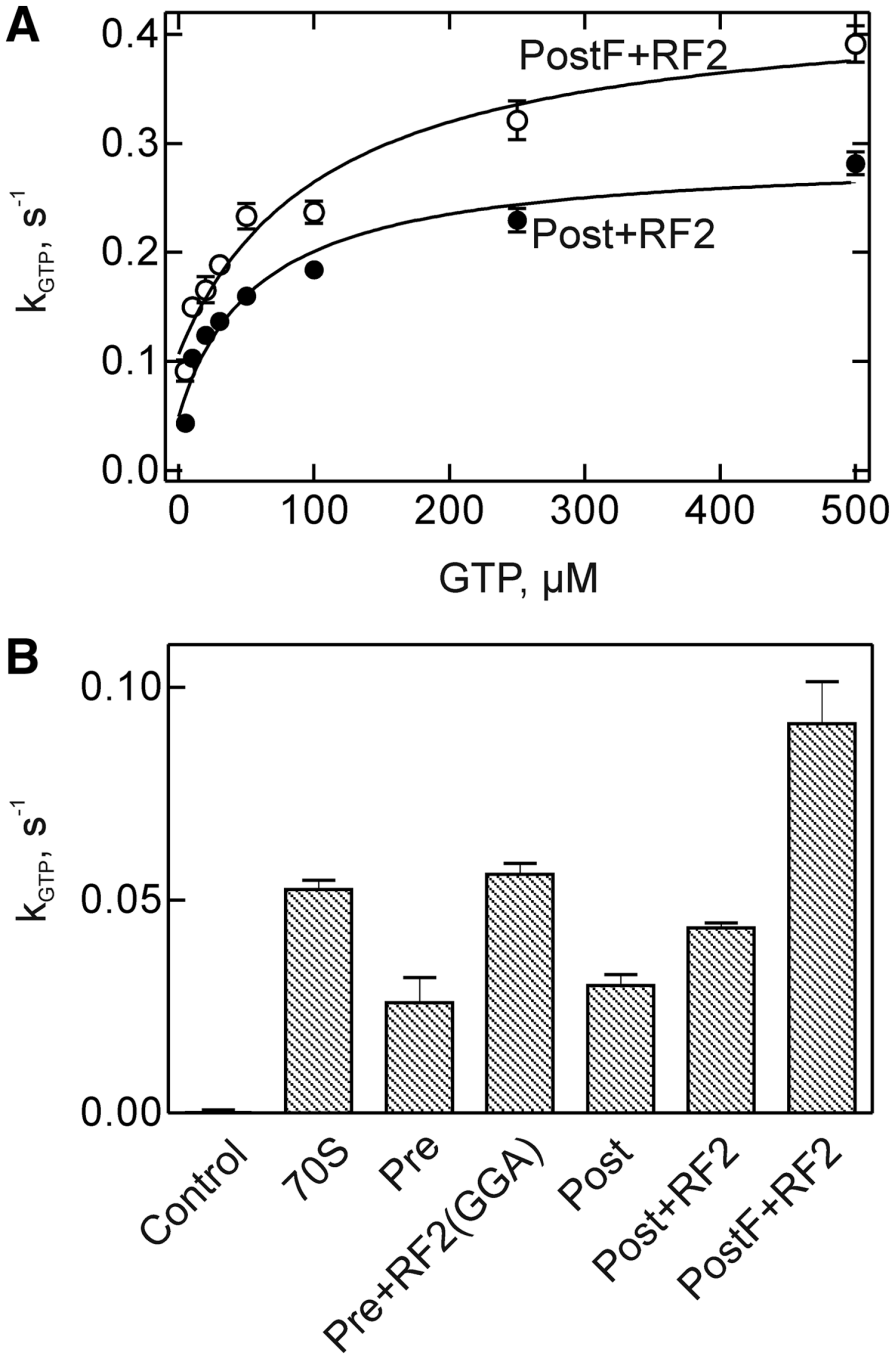
Kinetics of GTP hydrolysis by ribosome-bound RF3. **(A)** Dependence on GTP concentration. The hydrolysis of [γ-^32^P]GTP was measured with RF3 and PostTC with RF2 (obtained from PreTC containing fMet-tRNA^fMet^ in the P site by RF2 treatment), or PostTCF with RF2 (obtained from PreTC with fMetPhe-tRNA^Phe^ in the P site by treatment with RF2) (Methods). **(B)** GTP hydrolysis at low GTP concentration (5 µM; k_cat_/K_M_ conditions). Vacant ribosomes (70S), PreTC, PreTC with RF2(GGA), PostTC (obtained from PreTC by treatment with puromycin), PostTC with RF2, or PostTC with P-site tRNA^Phe^ (PostF) and RF2 as above; the control was performed with RF3 alone. Plotted is the initial velocity of GTP hydrolysis (k_GTP_) derived from time courses measured up to 60 s.

GTP was hydrolyzed by RF3 bound to the PostTC at a rate of ∼0.3–0.4 s^−^^1^ at saturation with GTP, close to the value published previously (17). The rate was not much different when either deacylated tRNA^fMet^ or tRNA^Phe^ was bound to the P site of the PostTC (Figure 5A). At low concentration of GTP, i.e. conditions where differences in catalytic rates and substrate binding are detected with high sensitivity (‘k_cat_/K_M_ conditions’), we observed that GTP was hydrolyzed at about the same rate (within a factor of two) when RF3–GTP was bound to vacant ribosomes, PreTC or PostTC (Figure 5B). The fact that GTP was hydrolyzed by RF3 bound to the PreTC clearly demonstrated GTP binding to that state. The GTPase rate was only slightly higher on the PostTC carrying tRNA^Phe^ in the P site, compared with the post complex with tRNA^fMet^, justifying the use of the latter complex as a model for studying RF3 function.

Furthermore, when RF2 was present, either as RF2(wt) on PostTC or as RF2(GGA) on PreTC, the activity of RF3 in hydrolyzing GTP was not changed much, up to 2-fold (Figure 5B). Thus, we do not observe the reported 4-fold stimulation by RF2 of the GTPase activity of RF3 on either PreTC or PostTC (17). This difference can be attributed to the fact that GTP hydrolysis previously was measured under conditions where >200 GTP molecules were hydrolyzed per RF3 present (17), whereas in our experiments, the extent of turnover was much less (2- or 3-fold). As nucleotide turnover on RF3, i.e. the dissociation of GDP following GTP hydrolysis, is strongly stimulated by RF1/2 (Figure 1), enhanced GTP hydrolysis measured under conditions of multiple turnover reflects the stimulation of turnover rather than of GTP hydrolysis itself.

## DISCUSSION

Our data confirm that the dissociation of GDP from RF3, which is relatively slow on free RF3, is accelerated when RF3–GDP is bound to the ribosome and RF2 is present (17,18). On the other hand, our data also show that GTP at equilibrium binds to RF3 with an affinity that is comparable with that of GDP (4-fold higher K_d_), rather than with a 500-fold higher K_d_, as reported previously based on data obtained by a non-equilibrium filtration assay (17). Given the small affinity difference between GDP and GTP for binding to RF3 (∼4-fold), a rate of spontaneous dissociation of GDP of ∼0.1 s^−^^1^ and an *in-vivo* GTP:GDP ratio of ∼10, we conclude that RF3 in the cell is predominantly present in the GTP-bound rather than the GDP-bound form, in contrast to Zavialov *et al.* (17), who proposed the GDP-bound form to be dominant and the only one to bind to the PreTC. Furthermore, we observe that RF3 binds and hydrolyzes GTP on both Pre- and PostTCs. This is in contrast to the proposal that GTP binding to RF3 bound to the PreTC, or the binding of RF3–GTP to the PreTC, was precluded and that GTP binding was restricted to RF3 bound to the PostTC (18). We note that the latter conclusions were based on small effects that are difficult to distinguish from the effect of RF1/2 on nucleotide turnover, in that observed GTP hydrolysis rates differed by factors of 2 to 4 only between PreTC and PostTC (18,19).

**Figure 6.**
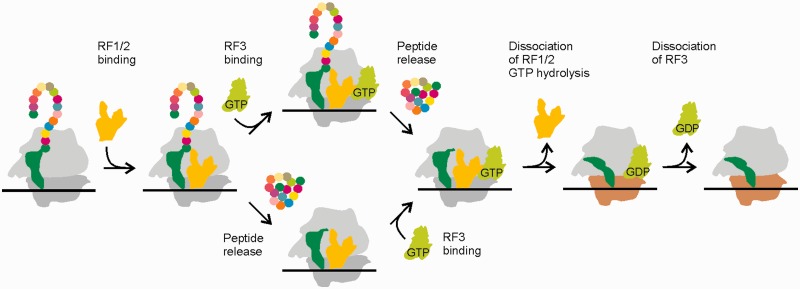
Schematic representation of translation termination. 50S ribosomal subunits are depicted in light gray, 30S subunits in dark gray or ochre to indicate different conformations. Peptidyl-tRNA is depicted in green, RF1/2 in yellow and RF3 in light green. The nascent peptide is depicted as colored balls. Binding of RF3**–**GDP and subsequent GDP-to-GTP exchange on RF3 as well as binding of RF3**–**GTP before RF1/2 binding is omitted.

Following peptide release, the affinity of GDPNP binding to RF3 is increased by three orders of magnitude owing to slower dissociation. The strong stabilization of the GDPNP-bound complex is in line with a closure of the nucleotide binding pocket that was derived from crystal structures of RF3 on the ribosome (11,12). The results of the GTPase assays imply that RF3 hydrolyzes GTP each time it binds to the ribosome, regardless of the functional state of the ribosome. This is in contrast to the proposal that the binding of RF3-GTP was excluded by peptidyl-tRNA in the P site and restricted to the post-termination state to avoid unproductive GTP hydrolysis (19). However, binding of RF3 to ribosomes that are not in the post-termination state is a rare event, as most of the time during protein synthesis, RF3 has to compete with the large excess of EF-Tu–GTP–aa-tRNA complexes, which is likely to preclude RF3 binding to the ribosome when a sense codon is exposed in the A site. Thus, GTP hydrolysis by RF3 on ribosomes that are in functional states other than the post-termination state is probably negligible. Present and previous (21) results suggest the following sequence of events during translation termination (Figure 6). Either RF1/2 binding to the PreTC is followed by peptide release and RF3–GTP binding to the PostTC (lower pathway in Figure 6); the binding of RF3–GDP and subsequent nucleotide exchange is also possible, but does not happen frequently due to the prevalence of the GTP-bound form of RF3. Alternatively, peptide release follows RF3–GTP binding to the PreTC (upper pathway in Figure 6). In both cases, peptide release is required before bound RF3–GTP can induce the hybrid/rotated state of the ribosome in which RF1/2 binding is destabilized (21). This way premature dissociation of RF1/2 on binding of RF3–GTP to the PreTC is minimized.

RF3 belongs to the family of translational GTPases, which also includes EF-Tu, EF-G, IF2, SelB and their eukaryotic orthologs (29). All these proteins have similar characteristic GTP binding motifs; however, they use the energy of GTP hydrolysis in remarkably different ways. While EF-Tu or SelB acts as GTPase-operated switches that are activated by codon–anticodon recognition (30,31), EF-G hydrolyzes GTP rapidly on binding regardless of the functional state of the ribosome (32–34) and uses the energy of GTP hydrolysis to promote directional tRNA movement (34). In comparison, ribosome-activated GTP hydrolysis by RF3 is slow, virtually independent on the functional state of the ribosome and does not require the presence of RF2. Rather, the timing of GTP hydrolysis appears to depend on the internal clock set by the structure of the GTP binding pocket and its interactions with the ribosome. The dramatic (1000-fold) difference in the GTP hydrolysis rates of EF-Tu/EF-G and RF3 may be explained by a markedly different orientation of the G domain of RF3 on the sarcin–ricin loop of the 50S subunit compared with that observed for EF-Tu and EF-G (12). The divergent evolution of translational GTPases and the mechanisms that cause the different rates of GTP hydrolysis are important issues to be addressed in the future.

## FUNDING

This work was supported by the Max Planck Society and a grant of the Deutsche Forschungsgemeinschaft (to M.V.R.). Funding for open access charge: Institutional.

*Conflict of interest statement*. None declared.
